# The Role of H3K4 Trimethylation in CpG Islands Hypermethylation in Cancer

**DOI:** 10.3390/biom11020143

**Published:** 2021-01-22

**Authors:** Giuseppe Zardo

**Affiliations:** Department of Experimental Medicine, School of Pharmacy and Medicine, University of Rome “Sapienza”, 00161 Rome, Italy; giuseppe.zardo@uniroma1.it

**Keywords:** H3K4 trimethylation, DNA hypermethylation, acute myeloid leukemia, cancer

## Abstract

CpG methylation in transposons, exons, introns and intergenic regions is important for long-term silencing, silencing of parasitic sequences and alternative promoters, regulating imprinted gene expression and determining X chromosome inactivation. Promoter CpG islands, although rich in CpG dinucleotides, are unmethylated and remain so during all phases of mammalian embryogenesis and development, except in specific cases. The biological mechanisms that contribute to the maintenance of the unmethylated state of CpG islands remain elusive, but the modification of established DNA methylation patterns is a common feature in all types of tumors and is considered as an event that intrinsically, or in association with genetic lesions, feeds carcinogenesis. In this review, we focus on the latest results describing the role that the levels of H3K4 trimethylation may have in determining the aberrant hypermethylation of CpG islands in tumors.

## 1. Histone Lysine Methylation

Epigenetic landscapes functionally define the chromatin architecture and they are shaped by the coordinated activity of “writers”, “readers” and “erasers”. “Writers” introduce covalent chemical modifications into DNA and histone tails, the “erasers” modulate the amount of these modifications and the “readers” recognize and bind the chemical modifications which induce functional effects in the chromatin architecture and DNA binding of transcription factors (TFs). Among the writers, histone methyltransferases catalyze the introduction of methyl groups in specific lysine and arginine residues at the amino terminal ends of the histone core [1], mainly at histones H3 and H4. Lysine methylation involves the ε-amine group of lysine at different positions of H3. Methylation events at K4, K9, K27, K36 and K79 are the most studied and characterized. Lysine can be mono-, di- or trimethylated. The level and state of histone lysine methylation depends not only on the activity of histone methyltransferases (KMTs) but also on the counteracting activity of histone lysine demethylases (KDMs). The variety of methylation sites and differentially methylated states describes the level of complexity of signaling mediated by histone lysine methylation, which is involved in transcription regulation, gene silencing, genome stability and RNA processing.

## 2. Histone Lysine 4 Methyltransferases

The enzymes responsible for histone lysine methylation (KMTs) contain a common active domain known as Su(var)3–9, Enhancer of zeste and Trithorax (SET), originally identified in yeast (SET1). Three SET1 homologs were subsequently identified in *Drosophila melanogaster*, including dSet1, Trithorax (Trx) and Trithorax-related [2], and 23 canonical SET-containing histone KMTs and one seven-beta-strand (7βS)-containing domain KMT (hDOT1L) with proven methyltransferase activity in mammals [3,4,5]. Some KMTs are highly selective. Each KMT methylates a specific lysine but not others located at different positions in the H3 polypeptide chain. For instance, the KMT that methylates H3K36 does not methylate H3K4, and the only KMT able to methylate H3K79 is hDOT1L [6,7,8,9,10]. In addition, a lysine can be specifically targeted by multiple enzymes. This redundancy allows specific activities to occur in a context-dependent manner. For instance, the same lysine may be modified by a different enzyme as a function of the histone’s genomic localization (enhancer versus promoter regions) but also to generate different methylation states (dimethylation versus trimethylation). KMT activity depends also on the specific lysine methylation state to add new methyl groups [11,12,13,14,15]. H3K4 methylation is one of the most studied and characterized histone lysine methylations. H3K4 can be mono-(H3K4me1), di-(H3K4me2) or tri-(H3K4me3). In mammals, H3K4 methylation is catalyzed by six SET domain-containing KMTs, namely SET1A/KMT2F, SET1B/KMT2G, MLL1/KMT2A, MLL2/KMT2B, MLL3/KMT2C and MLL4/KMT2D. Each of these enzymes is a component of multimeric complexes that may or may not contain other proteins such as WDR5, RbBP5, ASH2L and DPY30 [3]. These complexes are not redundant, as their activity marks H3K4 not only at functionally distinct loci but also at specific target genes determining different methylation states related to the recruitment of distinct “readers” [16,17]. For instance, multimeric complexes containing MLL1 and MLL2 trimethylate H3K4 at the promoter region of Hox gene clusters, which require the correct transcriptional regulation for hematopoietic development [18,19]. MLL2 is responsible for the tri-methylation of H3K4 of bivalent domains which is necessary for a mechanism aiming to maintain a paused transcriptional state in a targeted gene [20,21]. MLL3 and MLL4 monomethylate H3K4 located at the enhancer regions involved in cell type-specific gene expression [22,23,24,25]. Recent studies have revealed that the activity of KMT complexes is stimulated by the monoubiquitylation of histone H2B, and that distinct subunits components may have a role in determining the levels and state of H3K4 methylation [26,27,28].

## 3. Histone Lysine 4 Demethylases

To date, more than 30 KDM family members have been reported, and most of them contain a Jumonji domain, with the exception of KDM1A and KDM1B [29]. As for KMTs, KDMs target methylated lysines in H3, mainly at K4, K27, K9, K36 and K56, and in H4 at K20. KDMs demethylate specific lysines and not others located in different positions of the histone polypeptide chain. For instance, H3K27me3 is demethylated by KDM6B, which is not able to demethylate H3K4me3. KDMs may have distinct genomic localization and biological effects [30]. In mammals, H3K4 demethylation is catalyzed by the Jumonji, AT-rich interactive domain 1 (KDM5) and lysine-specific histone demethylase (KDM1) protein families. The KDM5 family is composed of four members designated KDM5A–D, and these enzymes are 2-oxoglutarate-dependent dioxygenases which require Fe^2+^ and O_2_ for their function in order to undergo the hydroxylation necessary to remove methyl groups [31]. All members contain conserved domains of five types: the ARID (DNA-binding domain), C5HC2 zinc finger, Jumonji C (JmjC), Jumonji N (JmjN) and plant homeodomain finger (PHD) (histone-binding domain) domains [32]. The KDM1 family is composed of the KDM1A member and its homolog, KDM1B, which are both Flavin Adenine Dinucleotide (FAD) -dependent histone lysine demethylases [33,34]. The KDM1A consists of three domains: the amine oxidase domain, the FAD binding domain and the SWIRM domain. In particular, the FAD binding domain consists of a Tower domain, which interacts with RE1-Silencing Transcription factor (REST), a transcription factor essential for demethylation activity [35]. KDM1B, however, does not bind REST [36,37]. KDM5A-D and KDM1A-B proteins have histone demethylases activity towards particular histone H3K4 methylation states; for instance, KDM5A demethylates H3K4me3/2 and processively H3K4me1, and KDM1 demethylates H3K4me1/2, with KDM1A also demethylating H3K9 [38,39,40,41,42,43,44]. KDM5A, KDM5C and KDM1A proteins form complexes with transcriptional repressors such as REST and KMTs establishing repressive chromatin marks [45,46]. Members of the KDM5 andKDM1 families may differ in their functions and biological effects. The KDM5A-D proteins are associated with transcriptional repression, as H3K4me3 is considered to be a transcriptional activating signal, since it is globally distributed, mainly at the promoters of the transcribed genes, and seems fundamental for recruiting the preinitiation factor Transcription Factor IID (TFIID) to certain gene promoters, even if loss of H3K4me3 does not always affect gene transcription [47]. However, KDM5A and B proteins may interact with different partners or complexes with transcriptional repressive functions such as Polycomb Repressive Complex 2 [46,48]. KDM5A interacts with the SIN3B-containing deacetylase and the nucleosome remodeling and deacetylase (NuRD) complexes [49]. KDM5B interacts with NuRD and KDM1A [50,51], whereas KDM5C interacts with the repressive H3K9 and H3K27 methyltransferase G9a in complex with histone deacetylases (HDACs) and REST [52]. Moreover, KDM5B protein may interact directly with HDACs mediating their recruitment to specific sites [53]. However, the activity of KDM5A–D also seems related, in some cases, to transcriptional activation, although it is not clear if this effect depends on demethylase activity or not [54,55]. As for the KDM5 protein family, KDM1 demethylase activity is also related to transcriptional repression. However, KDM1A, as it can demethylate H3K9, may be associated with transcriptional activation [56,57,58]. For instance, when KDM1A interacts with androgen and estrogen nuclear hormone receptors (AR and ER), it can demethylate H3K9me1/2, thus facilitating gene transcription [59,60]. Moreover, a neuron-specific isoform of KDM1An (also known as LSD1n) can target H3K20me2 controlling transcriptional elongation of a neuronal gene network [61]. Garcia-Bassets et al. [62] reported that 80% of the promoters occupied by KDM1A were bound to RNA polymerase II, suggesting that KDM1A was associated more often with active genes rather than the inactive genes. The formation of a protein complex including KDM1A, Rest corepressor (CoREST) and Growth factor independence (GFI) 1 proteins is also noteworthy [63]. This complex target represses a gene regulatory network that is necessary for normal hematopoiesis. KDM1A–GFI interaction may be disrupted by pharmacological molecules rescuing blast cell differentiation in acute myeloid leukemia with MLL translocations [64] and restoring the normal H3K4me3 state at targeted gene promoters. KDM1A is also found to be associated with long non-coding RNAs (LncRNAs) such as HOX Transcript Antisense RNA(HOTAIR), TElomeric Repeat-containing RNA (TERRA) and Steroid receptor RNA activator (SRA) [65]. Several non-histone proteins have been recognized as targets of KDM1A activity such as p53 [66], MYPT1 [67], E2F1 [68], and HIF-1α [69], which determine different effects on protein stability. JARID1 and LSD demethylases are involved in various cellular processes, including cell proliferation, embryonic mesenchymal transition, stemness, differentiation, cell motility, autophagy and senescence [70,71], and their dysregulation is also closely associated with embryonic development [72], human cancer development and other diseases [73].

## 4. K4 Methylated Histone H3’s Genomic Distribution and Function

The genomic distribution of methylated H3K4 has been studied both in simple eukaryotes, such as yeast, and in higher eukaryotes. In both cases, the distribution of methylated H3K4 is tightly associated with the state of methylation. In budding yeast, H3K4me3 localizes at gene promoter regions, H3K4me2 is mainly distributed within gene bodies and H3K4me1 tends to accumulate towards the 3’ end of genes [74]. In multicellular eukaryotes, genome-wide analyses of methylated H3K4 distribution show that H3K4me3 is predominantly localized at gene promoter regions, centered on the transcriptional start sites [75,76,77,78,79], while H3K4me2 tends to localize downstream of the H3K4me3 peak, and H3K4me1 is considered a marker of enhancer regions [80,81,82,83,84,85,86], although there is increasing evidence of H3K4me1’s role at gene promoters. H3K4me3 marks actively transcribed genes [75,87,88], in addition genes marked by the “broadest H3K4me3 domains” show increased transcriptional consistency [89]. Based on the observations described above, H3K4me3 has been proposed to sustain gene transcription [90,91]. However, the specific state of H3K4 methylation seems to have a role in involving distinct effectors regulating gene expression with different effects. For instance, Sims et al. [92] showed that the ATP-remodeling enzyme CHD1, which recruits the Spt-Ada-Gcn5 acetyltransferase (SAGA) complex [93] and sustains RNA polymerase II activity, recognizes H3K4me3. The bromodomain PHD finger transcription factor (BPTF), which is a component of the Nucleosome Remodeling Factor (NURF) complex, also recognizes and binds H3K4me3 [94]. Transcription factor IID (TFIID), through its PHD domain-containing TAF3 subunit, is recruited by H3K4me3, allowing more efficient preinitiation complex formation [95]. Several histone acetyltransferase-containing complexes such as SAGA, NuA3 and HBO1 may be recruited by H3K4me3 [96,97]. On the other hand, H3K4me2 was shown to bind to the Set3 complex. The Set3 complex induces histone deacetylation in 5’ transcribed regions. The resulting deacetylation slows gene activation. Set3C may repress internal cryptic promoters, but in different regions of genes from the Set2/Rpd3S pathway. In addition, Set3C induces transcription of some genes by repressing an overlapping antagonistic anti-sense transcript. Set3C histone deacetylase activity can combine with ncRNA transcription to delay or attenuate gene activation [98]. Although, as previously reported, H3K4me1 marks active enhancer regions when associated with H3K27ac, H3K4me1 at gene promoters has been shown to constrain the recruitment of H3K4me3-interacting reader proteins regulating the activity of corresponding genes [85].

## 5. H3K4me3 and H3K27me3 Overlapping: Genomic Distribution and Function

In the last two decades, the genomic distribution of modified histones has been extensively investigated, especially in relation to functionally distinct genomic compartments. The methodological approaches differ, and the continuous development of techniques capable of large-scale analysis has made it possible to obtain a precise picture of the distribution of modified histones in functionally different genomic regions. However, the functional consequences of these different distributions are still debated, so that more in-depth studies are necessary. For the purposes of this review, we will analyze the distribution and related functions of H3K4 and H3K27 methylation. In 2005, Bernstein et al. [81] and Kim et al. [99] conducted pioneering studies on genomic H3K4me2/3 distribution using human cancer cell lines and mouse fibroblasts cell lines. However, a year later, Bernstein et al. [100] observed that in mouse embryonic stem cells (mESCs), the majority of transcriptional start sites were found to be marked by H3K4me3. In the same study, they showed that H3K27me3 had a broader distribution but 75% of the H3K27me3 sites spanning transcriptional start sites (TSSs) were also marked by H3K4me3. These genomic locations were defined as bivalent domains. Genes that were marked by a bivalent domain at their 5’ end were found to be expressed at low levels, despite the presence of H3K4me3. Bernstein et al. also showed that by promoting differentiation toward the neuronal lineage, some bivalent genes became expressed and lost the H3K27me3 mark. These observations are fundamental to a model in which mainly developmental genes are marked by bivalent domains to pause or activate, or even permanently inactivate transcription as differentiation proceeds. Similar results were obtained in the work of Azaura et al. [101], which exploited replication timing as a surrogate for chromatin accessibility and the transcriptional status of genes. Mikkelsen et al. [102] combined the Chromatin Immunoprecipitation (ChIP) assay with next-generation sequencing (ChIP-seq) to analyze the genome-wide distribution of H3K4me3 and H3K27me3, and found that virtually all promoters with high CpG density (CpG islands) in mouse embryonic stem cells (ES) were marked by H3K4me3 and that a percentage of these promoters also exhibited H3K27me3. In addition, Mikkelsen et al. [102], and later Mohn et al. [103], showed that bivalent domains are not exclusive features of mouse embryonic stem cells (mESCs) but they also exist in differentiated cells, although to a lesser extent, as, during differentiation, bivalent domains are resolved in one direction or the other. Pan et al. [104], Zhao et al. [105] and Cui et al. [106] showed that bivalent domains were also present in cultured human embryonic stem cells (hESCs) and that the resolution of bivalency is required for lineage restriction [107,108]. However, stem cells in developing embryos are only transiently pluripotent, raising the question as to whether bivalency and other characteristics of embryonic stem cell chromatin are present in developing organisms as well. Bivalent domains also exist in pluripotent epiblast cells of early post-implantation embryos in mice [109]. However, one possibility is that bivalent domains might reflect the cellular heterogeneity of the samples analyzed rather than co-occurrence on individual nucleosomes or H3 histones. Several studies [104,110,111,112,113] suggest that bivalent domains exist and are not an artefact arising from culture conditions or heterogeneous samples.

Bernstein et al. [100] provided the first evidence that bivalent domains strongly correlate with CpG islands (CGI) in embryonic stem cells. CGI are a common feature of gene promoters in vertebrate genomes. They are defined by an elevated GC content, a ratio of observed to expected CpG dinucleotides of more than 0.6, 1 kb of length on average in promoter regions, and an unmethylated state in contrast to the methylated state of the vast majority of intragenic and intergenic CpG dinucleotides. Seventy percent of all promoters contain CGI [114,115,116], and all H3K4me3s mark CGI, whereas H3K27me3 show a broader distribution [102,104] with not all H3K27me3s marking CGI. These observations support the hypothesis that CGI may have a role in the establishment of bivalent domains. Artificially introduced CGI are able to recruit H3K4 and -27’s methylation activities [117]. The ability of histone K4 methyl transferases to target unmethylated CpGs depends on the presence of a CXXC domain or zinc finger CXXC (ZF-CXXC) DNA-binding domain. This domain characterizes MLLs [118,119], whereas it is absent in SET1A/B KMTs. However, in this case, the ability to target unmethylated CGI is mediated by a CXXC-containing protein called CFP1 (CXXC finger protein 1) [117,120,121], which is a component of SET1A/B KMT complexes. In addition, Eberl et al. [122] showed that the plant homeodomain finger (PHD) contained in CFP11 may target H3K4me3, favoring a mechanism that is able to sustain the accumulation of H3K4me3 at specific sites. An alternative mechanism for KMT recruitment to CGI is suggested by studies showing that Host cell factor 1, a component of KMT complexes, binds O-linked b-N-acetylglucosamine (O-GlcNAc) transferase (OGT) [123]. The transferase, in turn, interacts with the Ten–Eleven Trans-location (TET) family of proteins [124], which includes well-known players in maintaining the unmethyated state of CpG dinucleotides. Histone variants which are expressed throughout the cell cycle and deposited independently of DNA replication might also play a role in the establishment of bivalent marks at the gene promoter CpGi [125,126]. CGI may also guide the recruitment of the H3K27 methylating enzyme. In embryonic stem cells, H3K27me3-associated CGI are bivalent [102,127]. Polycomb repressive complex 2 (PRC2), through the EZH2 component, is responsible for H3K27 methylation. However, PRC2 components do not contain DNA-binding domains. Several authors [128,129,130,131,132,133,134] have shown that different proteins, such as Jarid2, PHF1 and MTF2, might be responsible for the recruitment of the PRC2 complex, and thus of EZH2, to GC-rich sequences. In addition, EZH2 and other PRC2 complexes interact with long ncRNAs [135] and short ncRNAs originating in proximity to or spanning CGI promoters [108,109,110,111,112,113,114,115,116,117,118,119,120,121,122,123,124,125,126,127,128,129,130,131,132,133,134,135,136].

## 6. CpG Dinucleotide: Unmethylated and Methylated Status

The human genome contains ∼29 million CpG dinucleotides, each of which may exist in the methylated or unmethylated state. Introns, 3′ untranslated regions and intergenic sequences are severely depleted in CpGs, whereas coding exons have a relatively higher density [137]. In contrast, CpG dinucleotides tend to form clusters in correspondence with mammalian gene promoters. These clusters are defined as CpG islands (CGI) and cover almost 75% of all genes, whereas the remaining 25% of genes are characterized as CpG-poor promoters. Whole genome methylation profiles [138,139,140] have shown that tandem and dispersed transposons tend to be heavily methylated, and exons, introns and intergenic regions tend to be heterogeneously methylated within a population of cells, while CpG-rich promoter regions are almost exclusively unmethylated in all tissue types.

CpG methylation in transposons, exons, introns and intergenic regions is important for long-term silencing, silencing of parasitic sequences and alternative promoters, regulating imprinted gene expression and determining X chromosome inactivation [141,142]. CGI, although rich in CpG dinucleotides, are unmethylated and remain so during all phases of mammalian embryogenesis and development, except in specific cases [143,144]. The biological mechanisms that contribute to the maintenance of the unmethylated state of CGI remain elusive, but the modification of established DNA methylation patterns is a common feature in all types of tumors and is considered as an event that intrinsically, or in association with genetic lesions, feeds carcinogenesis. In tumors, methylation events occur at promoter CGI, CpG shores, enhancers and insulators [145,146,147,148,149]. This aberrant DNA hypermethylation of such regulatory genomic regions is generally correlated with the repression of tumor suppressor [150,151,152,153], metastasis [154] and DNA repair genes [155,156], leading to the conclusion that DNA hypermethylation is directly responsible for the observed gene silencing [157,158,159]. However, recent experimental data on the characterization of the methylome of normal tissues and derived cancer types suggested that aberrant DNA hypermethylation represents a secondary event stabilizing gene inactivation, as most aberrantly hypermethylated genes in cancer are already repressed in the tissue of origin [160,161,162,163,164,165]. Nevertheless, DNA hypermethylation represents a mechanism directly affecting the expression of important tumor suppressor genes [166,167,168,169,170,171,172].

## 7. Mechanisms of the Protection of CpG Islands

**Figure 1:**
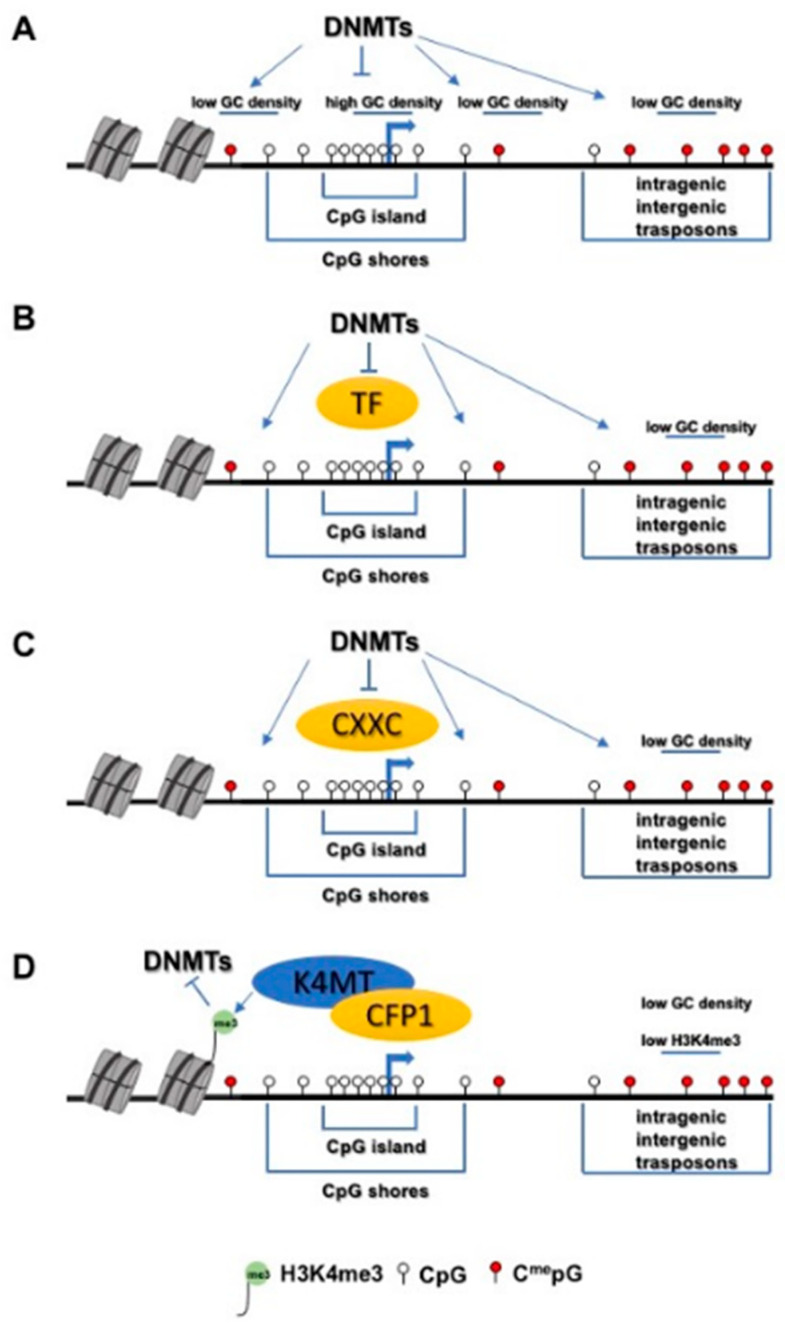
Schematic models of CpG islands protection. (**A**): effect of GC density and CpG frequency on de novo DNA methyltranserases (DNMTs); (**B**): effect of transcription factors on de novo DNMTs; (**C**): effect of CXXC domain containing protein on de novo DNMTs; (**D**): example of the effect produced by the recruitment oh histone H3K4 methyltransferases (K4MT) by CXXC domain containing proteins (CFP1) on the activity of de novo DNMTs.

The mechanisms that might generate a protective effect against CGI methylation are still not clear. The prevalent view is that the frequency of CpG dinucleotides and the content of C and G represent a key feature required to prevent methylation at CGI [117,173,174,175,176] (Figure 1). However, these observations must be integrated with others showing that specific transcription factor binding is linked to protection of the underlying DNA sequence [173,174,177,178,179,180] (Figure 1). Sequence features and specific transcription factor binding may also determine the recruitment of other proteins that might integrate their functions. In this view, all proteins containing a CXXC domain, such as CFP1, MLL1, MLL2, KDM2A, KDM2B, TET1 and TET3 [181] (Figure 1), which specifically targets them to CGI, might have a putative role in protecting CGI from methylation per se or because they recruit effectors able to block DNA methylation, triggering additional mechanisms. For instance, CFP1 recruits SET1A/B KMTs to CGI, which trimethylate H3K4, and trimethylated H3K4 has a specific role in inhibiting DNA methyltrasferases. H3K4me3 tends to be inversely correlated with DNA methylation [182,183,184]. The exclusive nature of this association of H3K4me3 with CGI methylation is related to its role in regulating DNA methyltransferase activity (Figure 1).

## 8. Mechanisms of CGI Hypermethylation in Cancer and the Role of H3K4me3

In cancer, CGI undergo methylation events defined as aberrant DNA hypermethylation. What causes the loss of the protective effect against methylation or, in other words, what interrupts a mechanism that appears to be based on sequence identity? It has been proposed that aberrant hypermethylation of CGI regions may be the consequence of the modified activity of DNA methyltransferases (DNMTs) [185,186,187,188,189,190,191]; aberrant recruitment of mutated transcription factors [192]; mutations in demethylating enzymes, such as ten-eleven translocation enzymes (TETs) and their associated cofactor pathways (isocitrate dehydrogenase; IDH1/2) [193,194,195,196,197]; and changes in chromatin architecture depending on post-translational histone modifications. Specific, global and local histone modifications are often associated with distinctive DNA methylation patterns [198]. Several studies investigating the molecular basis of DNA hypermethylation propose an “instructive mechanism” of aberrant DNA methylation in tumors that relies on histone modifications characterizing chromatin in embryonic and adult stem cells. Accordingly, CGI prone to hypermethylation in tumors are embedded in chromatin enriched in H3K27me3 only or H3K27me3 in association with an H3K4me3 mark at the same locus (bivalent domain) in human embryonic and adult stem cells [161,199,200,201]. This instructive model is supported by the observation that EZH1/2, a component of the PRC2 complex, is responsible for H3K27 methylation and may recruit de novo DNA methyltransferases (DNMTs) [202,203]. However, in hESCs as well as mESCs, H3K27me3 is mainly located in bivalent domains coinciding with unmethylated CGI [102,199,204,205]. This observation might indicate that H3K27me3 and DNA methylation are mutually exclusive. However, several independent studies have revealed a causal association among PRC2 recruitment, H3K27me3 and DNA hypermethylation during carcinogenesis [206,207,208,209]. A possible explanation for this apparent contradiction arises from the observation that TET1 and TET2 have been found to be associated with the PRC2 complex at CpGi in mESCs [210] and in cell lines overexpressing TETs 182]. TET enzymes catalyze hydroxylation of 5-methylcytosine and the active demethylation process, thus maintaining the unmethylated state of CGI.

**Figure 2:**
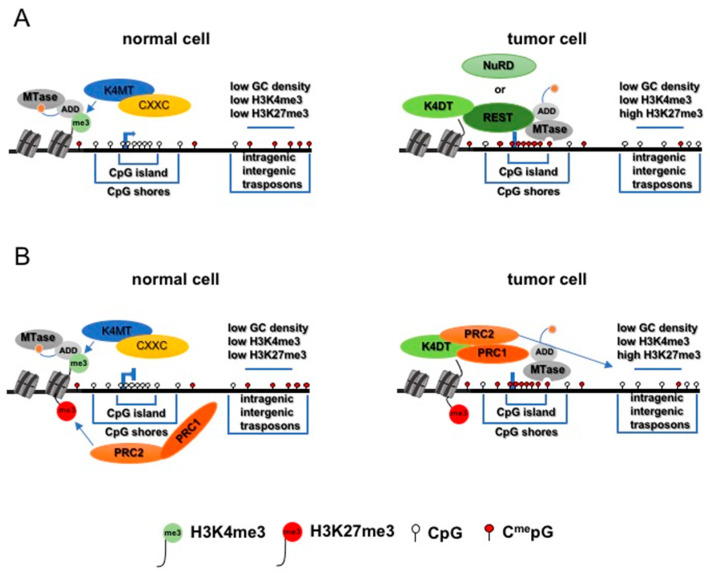
Effect of diminution of H3K4me3 levels on the activity of de novo methyltransferases (DNMTs) in tumor cell. (**A**): Promoter CpG islands marked by H3K4me3 in normal cells are protected by the H3K4me3 induced auto-inhibition of DNMTs. In tumor cell, the aberrant binding of repressive complexes (REST or NuRD) at promoter regions and the consequent recruitment of H3K4me3 demethylases (K4DT) causes the demethylation of H3K4me3 restoring the activity of de novo DNMTs and CpG island hypermethylation. (**B**): Promoter CpG islands marked by bivalent domains in normal cells are protected by the H3K4me3 induced auto-inhibition of DNMTs. In tumor cell, the aberrant recruitment of histone H3K4 demethylases (K4DT) by Polycomb Repressive Complexes (PRC2-PRC1) at promoter regions causes the demethylation of H3K4me3 restoring the activity of de novo DNMTs and CpG island hypermethylation.

Recent studies also indicate that methylation of CGI is related to the methylation status of H3K4 (Figure 2); the levels of methylated H3K4 (H3K4me3) tend to be inversely correlated with DNA methylation [183,184].

This mutually exclusive nature of the association of H3K4me3 with CGI methylation might be related to its role in regulating methyltransferase activity. The ATRX–DNMT3–DNMT3L domain (ADD) in the de novo DNA methyltransferase (DNMT) DNMT3a, for example, recognizes the unmethylated form of H3K4 (H3K4me0), which stimulates methyltransferase activity [211]. Through structural and biochemical analyses, the ADD domain of DNMT3a has been shown to also interact with its catalytic domain (CD), but in the presence of H3K4me3, DNMT3a loses the ability to bind and methylate DNA [212]. Thus, H3K4me0 and H3K4me3 have opposite effects on DNMT3a activity [213]. However, there is no evidence that an H3K4me3’s protective effect against aberrant methylation exists or is lost in tumors. Our studies [214] conducted on an acute myeloid leukemia cancer model suggest a direct role of the deregulation of H3K4me3 levels in determining the hypermethylation of the corresponding CGI.

In our study, we obtained, by Restriction Landmark Genome Scanning (RLGS) DNA methylation profiles of acute myeloid leukemia samples from patients and acute myeloid leukemia (AML) cell lines. We integrated methylation data with publicly available chromatin ChIP-seq data for H3K4me3 and H3K27me3 promoter CGI occupation in hESCs or hematopoietic stem and/or progenitor cells (hHSC/MPP). We observed that, in most cases, hypermethylated CGI in AML display H3K27me3 occupancy, even in the context of a bivalent domain in hESCs and hHSC/MPP. However, the analyses of specific hypermethylated CGI revealed a chromatin context characterized by a significant reduction in the H3K4me3 signal, with a concomitant increase in unmethylated H3K4 levels as opposed to a non-significant increase in the H3K27me3 mark, particularly in AML patient samples. Thus, we concluded that the loss of the normal levels of H3K4me3 in favor of increased levels of unmethylated H3K4 removes the de novo DNMTs auto-inhibition and promotes, as a consequence, aberrant CpGi hypermethylation. We also showed that the diminution of H3K4me3 levels is associated with the defective expression and recruitment patterns of specific writers, erasers and readers to CGI. Our proposal of the critical role of maintaining correct H3K4me3 levels to protect CGI from methylation was also suggested in recent works by Clarck [215] and Meehan [216]. Skvortsova [215] analyzed the pattern of H3K4me1 marked nucleosomes in embryonic stem cells and normal epithelial cells, and the mode of promoter CpG island hypermethylation in cancer. They observed that depletion or enrichment of H3K4me1 levels at the borders of CGI results in loss or gain of DNA methylation encroachment, and that H3K4me3, in contrast to H3K4me1 and H3K27me3, is highly enriched across the body of unmethylated CGI. In cancer cells, H3K4me3 is lost from DNA hypermethylated islands and is notably absent from the internal borders of the islands that undergo DNA methylation encroachment. Thus, relative enrichment of H3K4me1 and H3K4me3 represent a critical interface in predicting CGI DNA hypermethylation in cancer. The H3K4me1/H3K4me3 ratio was shown to be statistically significantly higher at CGI that become hypermethylated in cancer compared with CGI that remain unmethylated.

Moreover, H3K4me1-marked DNA exhibits low basal cytosine methylation in normal cells but a greater shift toward higher methylation levels in cancer cells in comparison with H3K27me3-marked DNA. On the other hand, Dunican et al. [216], using the concept of bivalent domains, revealed the existence in mESCs and hESCs of two subgroups of gene promoters, defined as HighBiv and LowBiv promoters, characterized by a high ratio of H3K27me3/H3K4me3 (enrichment in H3K27me3) and by a low ratio of H3K27me3/H3K4me3 (enrichment in H3K4me3), respectively. These two groups were associated with a different distribution of MLL2 that was enriched in LowBiv promoters, and with a different expression pattern showing that LowBiv promoters are highly expressed, although in the context of the typical reduced expression of bivalent domains. The analysis of DNA methylation levels in HighBiv and LowBiv regions in breast and colon cancer cell lines compared with normal cells showed that HighBiv promoters were more highly methylated than LowBiv promoters in cancer cell lines. In addition, they found little evidence of DNA hypermethylation at promoters that are characterized as H3K4me3-only or H3K27me3-only. Dunican et al. [216] suggested that a possible explanation for these results is that in LowBiv promoters, higher levels of H3K4me3 inhibit de novo methyltransferase activity. Dunican et al. [216] concluded that it could be “useful to investigate whether there is also a link between low level H3K4me3 and de novo DNA methylation at other discrete sets of CGI in cancer”. In this context, our study provides evidence that H3K4me3 levels play an important role in maintaining the unmethylated state of CGI in normal cells and that the H3K4me3-methylation-dependent protection of CGI is altered in tumor cells.

## 9. Conclusions and Perspectives

The modification of the H3K4 methylation state still represents an understudied event among the hypotheses describing how the unmethylated state of CGI is maintained in a normal cell or modified in a cancer cell, despite the fact that H3K4 methylation represents a powerful determinant of the activity of DNMTs 211-213]. To date, the connection between H3K4 trimethylation and the unmethylated state of CGI is derived from studies conducted with the aim of evaluating its genomic distribution [81,99,100,101,102,103,104,105,106]. Although these studies represent milestones in our understanding of the topography of the genomic distribution of H3K4 trimethylation, they do not represent a proof of concept of the role that H3K4 trimethylation may have in protecting CGI. There is no direct evidence that H3K4me3’s protective effect against aberrant methylation is lost in tumor cells. In our study, for the first time, to our knowledge, we demonstrated that in AML patient samples and AML cell lines, although based on a limited number of target CGI, DNA hypermethylation is associated with the loss of H3K4me3 and the acquisition of unmethylated H3K4. However, these results are not a proof of concept. Further studies need to be conducted to reveal, the genome-wide genomic distribution of unmethylated H3K4 and the significance of its association with hypermethylated CGI in cancer cells. In addition, a critical goal is to unveil the mechanisms underlying a change in the methylation state of H3K4 and the activity of DNMTs.
